# Announcment: Gordon Research Conference “Metals in Biology”

**DOI:** 10.1155/MBD.1995.293

**Published:** 1995


					TA IN BIOLO
ORDON EARCH CONFERE
PROGRAM
January 21-25, 1996
Doubletree Hotel, Ventura, CA, USA
A Look to the Future, Richard Holm, Harvard University, USA: Chair
Gregory Petsko, Brandeis University, USA: Cytochrome P450 in Four Dimensions
Cytochrome Oxidases, Gerald Babcock, Michigan State University, USA: Chair
Robert Gennis, University of Illinois, USA: Structure and function of the heine-copper oxidases
Ninian Blackburn, Oregon Graduate Institute, USA: XAS studies on heine-copper oxidases and
models
Stuart Ferguson, Oxford University, United Kingdom: Structure of Cytochrome cdl: An Oxidase
and a Nitrite Reductase with an Unusual Haem
Models for Oxidases, Kenneth Karlin, Johns Hopkins University, USA: Chair
James Collman, Stanford University, USA: Functional Synthetic Analogues of the Oxygen
Binding/Activating Heine Proteins: Myoglobin and Cytochrome c Oxidase
Joann Sanders-Loehr, Oregon Graduate Institute, USA: Raman spectroscopy of blue, non-
blue, and purple copper proteins
William Tolman, University of Minnesota, USA: Using synthetic chemistry to gain insight into O-O
bond cleavage and C-H bond activation reactions of copper proteins
Metal DNA Processing and Repair, Thomas O'Halloran, Northwestern University,
USA: Chair
Gregory Verdine, Harvard University, USA: Molecular mechanism of the Ada protein: a
metalloactivated chemosensor for methylation damage in DNA
Jacqueline Barton, California Institute of Technology, USA: Damage and repair of DNA by
rhodium complexes
Dagmar Ringe, Brandeis University, USA:The structure of the iron-activated regulatory protein
diphtheria tox repressor
Metal Channels and Neuroscience Signal Transduction Jeremy Berg,
Johns Hopkins University, USA: Chair
Joseph Falke, University of Colorado, Boulder, USA: Molecular tuning of calcium binding sites in
signaling pathways
Gary Yellen, Massachusetts General Hospital, USA: A conformation-sensitive engineered metal
site in an ion channel
Metal-binding Biomolecules and Metallorecognition, John Groves,
Princeton University, USA: Chair
Francois Diederich, ETH-Zurich, Switzerland: Dendritic metalloporphyrins and dendritic
receptors
Carol Fierke, Duke University Medical Center, USA: Architecture of catalytic zinc binding sites in
proteins
Claude Meares, University of California, Davis, USA: Mapping protein surfaces with metal ions
Metal Mobilization, Edward Stiefel, Exxon, USA" Chair
Donald Kurtz, Jr., University of Georgia, USA: Structure and redox properties of rubrerythrin
Peter Lindley, CCLRC Daresbury Laboratory, United Kingdom: The X-ray structure of human
ceruloplasmin at 3.1 A: a putative role for the enzyme in iron metabolism
293

				

